# Loss of Control Eating in Adults With Impulsive and/or Inattentive Tendencies

**DOI:** 10.1002/brb3.70092

**Published:** 2024-10-08

**Authors:** Laura Christie, James H. Smith‐Spark, Rachel D. Teodorini

**Affiliations:** ^1^ Division of Psychology London South Bank University London UK

**Keywords:** adult attention deficit hyperactivity disorder (ADHD), disordered eating, impulsive, inattentive, loss of control eating

## Abstract

**Objective:**

Previous research has tended to consider impulsive, inattentive, and loss of control eating (LOC) tendencies as symptoms of greater pathologies in treatment‐seeking samples. However, inattentive and impulsive tendencies and LOC often co‐occur. Although LOC is an important diagnostic component of disordered eating (ED), it has recently been argued to be a dysregulated eating behavior in its own right. The purpose of the current self‐report study was, therefore, to investigate the association between impulsive and inattentive tendencies and LOC in adults after accounting for ED.

**Method:**

A community sample of 516 adults was surveyed online about their inattentive and impulsive tendencies, LOC, and ED behaviors.

**Results:**

A hierarchical multiple linear regression revealed ED, inattentive, and impulsive symptoms to be independent, significant, positive predictors of LOC.

**Discussion:**

These findings suggest that the levels of inattentive and, to a lesser extent, impulsive tendencies are significantly associated with LOC in adults, even after ED is accounted for. Moreover, inattentive tendencies were found to be more significantly associated with LOC than impulsive tendencies. These are novel and important findings that can be used to inform both clinicians and individuals with inattentive and impulsive tendencies alike of this association. Considering the well‐documented adverse health and wellbeing outcomes associated with LOC, future feasibility trials are needed aimed at treating this co‐occurrence.

## Graphical abstract

This study investigated the relationship between loss of control eating (LOC) and levels of inattentive and impulsive tendencies in adults. A sample of 516 adults was surveyed online about their inattentive and impulsive tendencies, LOC, and disordered eating (ED) behaviors. Findings revealed that levels of inattentive and, to a lesser extent, impulsive tendencies are significantly associated with LOC in adults, even after ED is accounted for. Inattentive tendencies were found to be more significantly associated with LOC than impulsive tendencies.

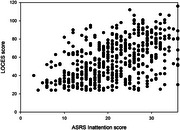

## 1 Introduction

Loss of control eating (LOC) is defined as a subjective lack of agency over one's eating behavior (Goldschmidt 2017). It is prevalent in the United States, Europe, Oceania, and Asia (He et al. 2017) and currently presents in 6%–10% of adults (Goldschmidt et al. 2015; Solmi et al. 2014). LOC eating is associated with psychiatric co‐occurrences, obesity, reduced social functioning, and quality of life (Dalle Grave, Calugi, and Marchesini 2012; Jenkins et al. 2012). It is also a core element of binge eating disorder (BED) (Telch, Pratt, and Niego 1998), characterized by the consumption of large quantities of food over a short period of time (Giel et al. 2022). Although LOC can be distinguished from BED by the individual's cognitions, subjective experience, and psychological distress during eating episodes (Bartholome et al. 2006), to the authors’ best knowledge, there is no diagnostic measure of caloric intake that distinguishes BED from LOC. However, Kurz et al. (2017) have argued that LOC is closely linked with the attention deficit hyperactivity disorder (ADHD) symptoms of inattention and impulsivity. Impulsivity is defined as an immediate reaction to stimuli without adequate consideration of their consequences and conscious judgment (Bakhshani 2014). Inattention is defined as a failure to maintain focus or attend to the task at hand (Palmer and Finger 2003). This putative relationship suggests that ADHD symptomology could be a means by which to distinguish LOC from BED. Establishing such a relationship could aid in preventing the progression of LOC to BED in such cases. The current study, therefore, aimed to investigate whether levels of inattention and impulsivity could predict LOC independently from BED.

In 2020, the prevalence of adult ADHD was reported as being 6.76% of the world population (Song et al. 2021). Although adult ADHD co‐occurs with a number of psychiatric conditions (Katzman et al. 2017; Mohammadi et al. 2021), the co‐occurrence of impulsivity and inattention with LOC has been overlooked (Cortese and Vincenzi 2011). The rate of LOC behaviors in adults with inattentive and impulsive tendencies is higher than in the general population (Kaisari et al. 2018; Svedlund et al. 2017, 2018). Studies have identified significant positive associations between inattentive and impulsive symptoms and LOC (Kaisari et al. 2018; Seitz, Kahraman‐Lanzerath, and Legenbauer 2013; Svedlund et al. 2017, 2018). Svedlund et al. (2017) assessed adult ED patients for inattentive and impulsive symptoms, ED symptoms, and co‐occurring mental health problems. Disordered eating (ED) is defined as being a severe disturbance in one's eating behaviors and related thoughts and emotions due to psychosocial cognitive distortions and emotional motivations towards food, one's body shape, and/or weight (Davidson et al. 2022). Svedlund et al. found a significant positive correlation between the patients’ self‐reported inattentive and impulsive scores and LOC, highlighting the high frequencies of inattentive and impulsive symptoms in patients with LOC, with this relationship being as strong as that found in patients with BED and disorders characterized by LOC. Rather than LOC simply being a symptom of BED/ED, inattentive and impulsive tendencies were associated with LOC uniquely. Furthermore, Svedlund et al. observed that inattention was more prominently associated with LOC than hyperactivity/impulsivity in female adult ED patients.

There are several reasons why inattentive and impulsive tendencies and LOC may co‐occur. It might be that LOC co‐varies with both inattentive and impulsive symptoms (Svedlund et al. 2017). Inattention can lead to poor awareness of internal cues of hunger and satiety (Flemings et al. 2005; Kaisari et al. 2018), as well as difficulties in goal‐directed food choices and eating behavior (Ammar et al. 2020; Davis et al. 2006). These can then contribute to LOC (Kaisari et al. 2018; Lanoye, Adams, and Fuemmeler 2022). However, impulsivity could also cause ill‐considered actions, such as eating in response to low mood or anxiety (Crockett, Myhre, and Rokke 2015; Waltmann et al. 2021) and emotional eating (Ferrell, Watford, and Braden 2020), resulting in high‐caloric food choices that can lead to LOC (see Seymour et al. 2015 for a review). That said, Martin, Dourish, and Higgs (2023) found that interoceptive accuracy mediates the relationship specifically between inattention and binge‐type eating. Interoceptive accuracy refers to the ability to accurately receive internal signals relating to fullness and satiation. If an individual has inattentive tendencies, it is likely that they have poor interoceptive accuracy and, thus, poor awareness of internal cues of hunger and satiety. Therefore, inattention may be more significantly related to LOC than impulsivity.

The emerging evidence is, therefore, that LOC, or ED marked by LOC (such as BED), occurs at higher‐than‐expected rates in people with inattentive and impulsive tendencies (Kaisari et al. 2018; Svedlund et al. 2017, 2018). Further to this, the same pharmaceutical medication, lisdexamfetamine, is prescribed for both BED and ADHD (Childress et al. 2022; Schneider, Higgs, and Dourish 2021), again suggesting that LOC or ED marked by LOC (such as BED) is associated with inattentive and impulsive tendencies. However, because previous research has assessed associations between inattentive and impulsive tenancies and LOC, the current understanding of the independent associations between LOC and inattentive and impulsive symptoms is limited.

Moreover, the presence (or recovery stage) of ED is a confounding variable when looking at LOC independently from BED (Hornberger et al. 2021). As LOC is a symptom of ED, it includes some of the same cognitive distortions, such as “while eating, I feel disgusting” (Latner et al. 2014). However, cognitive distortions in LOC are distinguished from those of ED; in the latter case, the psychological distress of cognitive distortions is not limited to behavior‐based episodes (Fairburn, Cooper, and Shafran 2003). Instead, merely thinking about certain foods can increase self‐perceptions of “fatness” (Coelho et al. 2013). Latner et al. (2014) have argued that this pattern of thinking would not occur in a sample with LOC alone. Therefore, it can be argued that LOC should be considered independently of ED in relation to ADHD symptomology.

As cognitive distortions have been argued to occur in ED but not in LOC alone (Latner et al. 2014), the current study aimed to investigate the association between inattentive and impulsive traits and LOC independently. Therefore, ED was accounted for in the current sample. It was hypothesized that (1) the level of self‐reported impulsivity would be a significant positive predictor of LOC after accounting for ED (Kaisari et al. 2018; Svedlund et al. 2017, 2018) and (2) the level of self‐reported inattention would be a significant positive predictor of LOC after accounting for ED and impulsivity (Mikami et al. 2010; Svedlund et al. 2017, 2018).

## Methods

### Respondents

A total of 516 respondents were recruited via online advertisements on the Reddit (http://www.reddit.com) online forum site. The mean age of the sample was 33.78 years (SD = 11.90, range = 18–80 years). A total of 183 respondents reported as male, 312 reported as female, 16 respondents reported as non‐binary, 1 reported as a transgender woman, 1 reported as a gender, and 3 respondents preferred not to say. No reward was offered for participation. To be included in the study, respondents had to be aged between 18 and 80 years and not in treatment for addictions at the time of the study, nor diagnosed with ED or currently receiving therapy. The survey was conducted between June 11 and June 30, 2022.

### Materials

Qualtrics XM survey software was used to create the online survey. The 18‐item ASRS (Kessler et al. 2005) was used to measure the frequency of self‐reported inattentiveness and impulsiveness over the previous 6 months. The 18 items reflect the 18 DSM‐IV, Text Revision criteria for ADHD. The ASRS assessed the level of inattentive and impulsive tendencies through the use of two subscales. Questions 1–3 and 6–13 probed inattentiveness (e.g., “How often do you have difficulty getting things in order when you have to do a task that requires organization?”), whereas Questions 4, 5, and 14–18 measured impulsivity (e.g., “How often do you interrupt others when they are busy?”). Items were scored on a 5‐point scale (0 = never, 1 = rarely, 2 = sometimes, 3 = often, 4 = very often), with higher scores indicative of greater severity of reported inattentiveness and impulsiveness. Although the ASRS was designed for diagnostic purposes in clinical contexts, the current study looked at behavioral patterns rather than diagnostic thresholds. Scores for the two subscales of inattention and hyperactivity were added together to produce a score ranging from 0 to 36 for each subscale. On both subscales, the higher the score, the greater the respondent's self‐rated inattentive or impulsive tendencies. Cronbach's alpha for the ASRS in the current study was excellent (*α* = 0.91).

The original 24‐item Loss of Control Eating Scale (LOCES; Latner et al. 2014) was designed to measure the sense of LOC in clinical and non‐clinical samples. In the current study, it was used to measure the frequency of a variety of self‐reported behaviors (e.g., “I felt I was eating faster than normal”), cognitions (e.g., “While eating, I felt like I was in my own little world”), and positive/euphoric feelings (e.g., “While eating, I felt a physical rush or high”) experienced while eating over the last 28 days. Items were scored on a 5‐point Likert scale (1 = never, 2 = rarely, 3 = occasionally, 4 = often, 5 = always) and were summed to give an overall score, with higher scores indicative of greater severity of LOC. The Cronbach's alpha for the LOCES in the current study was also excellent (*α* = 0.96).

The five‐item self‐report SCOFF Questionnaire (Morgan, Reid, and Lacey 1999) presented five statements indicative of ED behavior (e.g., had made themselves sick because they felt uncomfortably full) and cognitions (e.g., believed themselves to be fat when others said that they were too thin), asking the participant to select “yes” or “no” answers to accurately reflect their experience over the last year. The SCOFF Questionnaire is not a diagnostic instrument but has been found to have high specificity and sensitivity in identifying individuals with symptoms that are highly consistent with ED (Luck et al. 2002). Thus, for the purposes of the current study, this identification of symptoms highly consistent with ED is referred to as ED. In the current study, answers of “no” and “yes” were given scores of “1” and “2,” respectively. These responses were summed to yield a final score between 5 and 10, with higher scores being indicative of a greater severity of ED. Cronbach's alpha for the SCOFF Questionnaire in the current study was moderate (*α* = 0.51); the violation of tau‐equivalence is explained by the low number of items making up the SCOFF (Hinton, McMurray, and Brownlow 2014).

### Design

A correlational design was used to investigate the relationship between inattentive and impulsive tendencies after accounting for ED. The data were analyzed using hierarchical regression. The predictor variables were ED (as assessed by the SCOFF), impulsivity, and inattention (both measured by the ASRS). For the hierarchical regression, ED was entered as a predictor in Block 1, impulsivity was entered in Block 2, and inattention was entered in Block 3. The outcome variable was the level of LOC as assessed by the LOCES.

### Procedures

The study was approved by the School of Applied Sciences Research Ethics Committee (ETH2223‐0015) at London South Bank University. The link to the online questionnaire was advertised on several sub‐Reddit forums. Clicking on the link brought respondents to the information sheet, followed by the consent form, and once clicked, indicating consent, the questionnaire was then presented. The respondents were required to provide demographic information on age and gender. They then completed the questionnaires in the following order: ASRS, LOCES, and SCOFF. The respondents who identified themselves as taking ADHD medication were asked to answer all questionnaires true to when they were unmedicated. The respondents were then debriefed in writing once all questionnaires had been completed. It took no more than 15 min for the respondents to complete the study.

## Results

Descriptive statistics for the predictor variables and criterion variable are detailed in Table 1.

**TABLE 1:** Descriptive statistics for predictor and criterion variables.

	Minimum	Maximum	Mean (SD)
Total ASRS score	5	10	8.67 (1.25)
Inattentive score	3	36	21.31 (7.38)
Impulsive score	0	36	16.81 (7.10)
SCOFF score	5	10	8.67 (1.25)
LOC score	24	116	57.11 (21.15)

Abbreviation: LOC, loss of control eating.

Bivariate correlations are displayed in Table 2. ED had a strong positive, significant bivariate relationship with LOC. Impulsivity was found to have a moderately positive significant bivariate relationship with LOC. Inattention had a moderately positive significant relationship with LOC.

**TABLE 2:** Bivariate correlation matrices for disordered eating (ED), loss of control eating (LOC), impulsivity, and Inattention.

	1	2	3	4
1. LOC	—	0.69^***^	0.47^***^	0.47^***^
2. ED		—	0.34^***^	0.33^***^
3. Impulsivity			—	0.61^***^
4. Inattention				—

*** *p *< 0.001.

**FIGURE 1:**
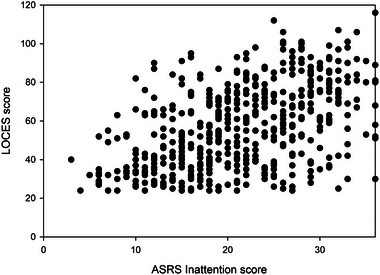
The relationship between LOC and inattention. LOC, loss of control; LOCES, loss of control eating scale.

**FIGURE 2:**
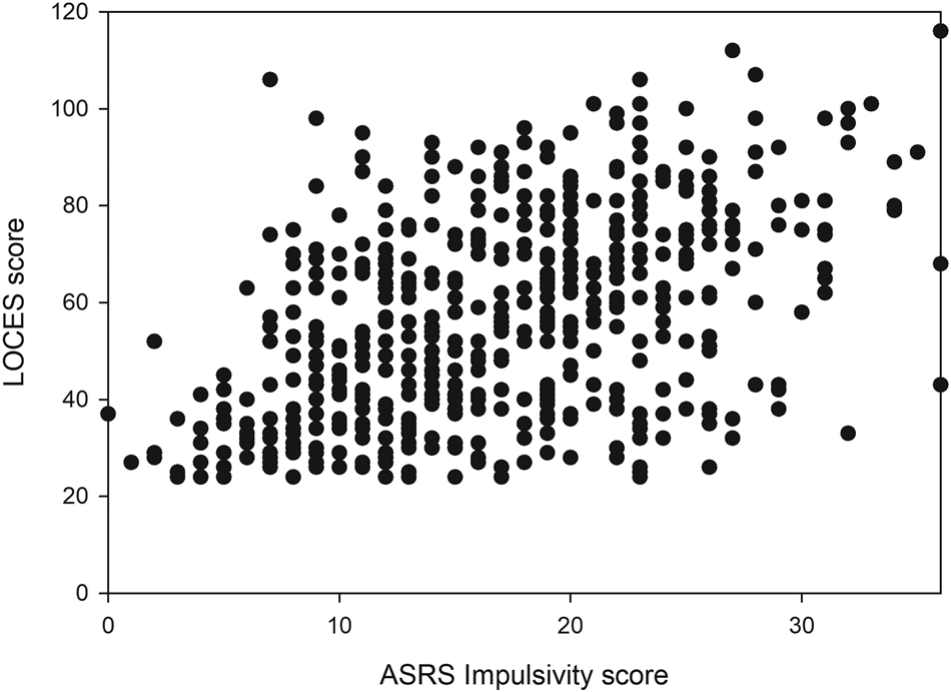
The relationship between LOC and impulsivity. LOC, loss of controle eating; LOCES, loss of control eating scale.

Figures 1 and 2 show the relationships between LOC and inattention and impulsivity respectively.

A hierarchical multiple linear regression was carried out to determine whether ED, impulsivity, and inattention would predict LOC. The results are shown in Tables 3 and 4.

**TABLE 3:** Model summary for a three‐stage hierarchical regression of disordered eating (ED), impulsivity, and inattention as predictors of loss of control (LOC).

	Adjusted *R* ^2^	*R* ^2^ change	Model ANOVA results	*R* ^2^ change ANOVA results	Standard error estimate
Block 1 (ED)	0.48	N/A	*F*(1, 514) = 473.35, *p* < 0.001	N/A	15.28
Block 2 (ED; impulsivity)	0.54	0.06	*F(*2, 513) = 300.22, *p* < 0.001	*F* change (2, 513) = 66.64, *p* < 0.001	14.39
Block 3 (ED; inattention; impulsivity)	0.56	0.02	*F*(3, 512) = 215.29, *p* < 0.001	*F* change (3, 512) = 21.47, *p* < 0.001	14.11

**TABLE 4:** Coefficients for a three‐stage hierarchical regression of disordered eating (ED), impulsivity, and inattention as predictors of loss of control eating (LOC).

	Unstandardized B	Unstandardized SE	*t*	*p*	Standardized beta coefficient
Block 1					
Constant	− 17.09	3.48	− 4.92	< 0.001	
ED	11.72	0.54	21.76	< 0.001	0.69
Block 2					
Constant	− 20.61	3.30	− 6.24	< 0.001	
ED	10.22	0.54	18.94	< 0.001	0.60
Impulsivity	0.78	0.10	8.16	< 0.001	0.26
Block 3					
Constant	− 23.84	3.31	− 7.20	< 0.001	
ED	9.87	0.54	18.47	< 0.001	0.58
Impulsivity	0.42	0.12	3.42	0.001	0.14
Inattention	0.54	0.12	4.63	< 0.001	0.19

In Block 1, ED was found to be a significant predictor of LOC, accounting for 48% of the variance. When impulsivity was entered in Block 2, there was a significant change in the ability to predict LOC. Impulsivity was found to explain an additional 6% of the variance in LOC. When inattention was entered in Block 3, there was a significant change in the ability to predict LOC. Inattention accounted for an additional 2% of the variance in LOC.

ED, impulsivity, and inattentive symptoms were all significant independent predictors of LOC, with this pattern being found in all blocks. All significant predictors had positive relationships with LOC. Table 4 shows the coefficient summary.

## Discussion

The current online self‐report study accounted for ED before testing the association between inattentive and impulsive tendencies and LOC in adults. In finding that inattentive and impulsive tendencies were both independent, significant, and positive predictors of LOC, the results supported the hypotheses and were also consistent with the previous literature (Kaisari et al. 2018; Mattos et al. 2004; Mikami et al. 2010; Seitz et al. 2013; Svedlund et al. 2017, 2018). Past research has found that the ADHD symptoms of impulsivity and inattention are significant positive correlates with ED as marked by LOC, such as BED (Neumark‐Sztainer et al. 1995; Seitz et al. 2013). Such studies drew conclusions that associated ADHD with ED, such as BED. Because it controlled for ED, the current study extended previous work by demonstrating the significant, positive association between inattentive and impulsive tendencies and LOC uniquely, independent of ED symptomology. The current study extended the previous literature in indicating that the level of inattention and impulsiveness are significantly associated with LOC even after accounting for ED. These novel findings support the relationship between inattentive, impulsive, and LOC tendencies independent of clinical diagnoses (such as BED). Additionally, these findings highlight an area requiring greater awareness in order to prevent and reduce the prevalence of this behavior and the progression of the individual to ED.

Moreover, the current study provides further evidence of inattentive (rather than impulsive) tendencies as being more significantly associated with LOC (Seitz et al. 2013; Svedlund et al. 2017, 2018). This is an important finding and has implications not only for those diagnosed with ADHD of the primarily inattentive type but also for undiagnosed individuals who struggle with inattention and those with subclinical levels of inattention. Furthermore, these findings have implications for clinical treatment and maintenance tools (Kaisari et al. 2018; Mattos et al. 2004; Mikami et al. 2010; Seitz et al. 2013; Svedlund et al. 2017, 2018). The current treatment options for adults are limited to types of cognitive behavioral therapy, and these could be significantly impaired by inattentive and impulsive symptoms (Carlucci et al. 2017). Therefore, the development of clinical treatment and maintenance tools particularly for those with inattentive and LOC comorbidities seems warranted. Additionally, this stronger association with inattention may be due to interoceptive accuracy (Martin, Dourish, and Higgs 2023), and this too has implications for the development of screening tools specifically for those with inattention and those at risk for eating disorders. Regardless of the nature of the relationship between inattention and LOC, LOC has been found to be associated with adverse physical health consequences (Kelly, Cotter, and Guidinger 2018), lower quality of life (Jenkins et al. 2012), and greater impairment in social and working life (Gralle et al. 2017; Pawaskar et al. 2017).

There are a number of strengths to the present study. Although it targeted adults only, the current study looked at groups with inattentive and impulsive tendencies separately. The current study also sampled participants online. By doing so, participation was made accessible to a wide demographic through posting on various forums. Online surveys provide the kind of anonymity that in‐person data collection methods are unable to offer to the respondent, and this anonymity may reduce social desirability effects (Joinson 1999). Therefore, reporting levels of impulsivity, inattention, and LOC may be more reliable via online methods rather than studies using in‐person self‐report assessments (Seitz et al. 2013; Svedlund et al. 2017, 2018).

A limitation of the current study was that it did not control for related disorders of both inattentive and impulsive tendencies and LOC, such as depression (Colles, Dixon, and O'Brien 2008), anxiety (Rosenbaum and White 2015), and personality disorders (Becker et al. 2010; Farstad, McGeown, and von Ranson 2016; Herzog et al. 1992; Wonderlich et al. 1990). However, to do so would have greatly increased the length and completion time of the survey, which would have likely resulted in higher drop‐out rates. Thus, a fundamental unanswered question that should be addressed in future research is the extent to which LOC is specific to inattentive and impulsive tendencies or whether the construct represents more generalized pathology or transdiagnostic constructs, such as impaired emotional regulation (Davis et al. 2006; Goldschmidt et al. 2017; Lee‐Winn et al. 2016). It is also unclear whether the experience of LOC is similar across pathologies, as noted in previous studies (Kaisari et al. 2018). This lack of clarity highlights the importance of collaboration among specialists in answering this question, as the use of different labels to describe similar experiences limits the generalizability of findings from research looking at similar behavioral problems.

To conclude, the findings of the current study suggest that the level of inattentive (and, to a lesser extent, impulsive) tendencies among a diverse adult sample is significantly associated with LOC, even after accounting statistically for ED. Considering the well‐established adverse health and wellbeing outcomes associated with LOC (Gralle et al. 2017; Jenkins et al. 2012; Kelly, Cotter, and Guidinger 2018; Pawaskar et al. 2017), early identification of LOC and treatment interventions are essential (Arango et al. 2018). Therefore, the creation of clinical tools to help those with inattentive tendencies and LOC comorbidities is warranted in the early assessment stages. The current study has also demonstrated that inattentive and, to a lesser extent, impulsive tendencies are significantly associated with patterns of LOC unrelated to ED. Clinicians, individuals with ADHD, especially the inattentive type, and undiagnosed individuals with inattentive tendencies need to be aware of this association so that steps can be taken to prevent or reduce the prevalence of LOC in this population.

## Author Contributions

**Laura Christie**: conceptualization, data curation, formal analysis, investigation, methodology, project administration, software, writing–original draft. **Rachel D. Teodorini**: methodology, software, supervision, writing–review and editing. **James H. Smith‐Spark**: writing–review and editing.

### Peer Review

The peer review history for this article is available at https://publons.com/publon/10.1002/brb3.70092.

## Public Significance Statement

The findings of this study are important in informing people who struggle with inattention and impulsivity about the possible tendency to lose control of eating. This is also an important finding to inform clinicians of this association.

## Data Availability

Data available on request from the authors.
